# Craniospinal irradiation(CSI) in patients with leptomeningeal metastases: risk-benefit-profile and development of a prognostic score for decision making in the palliative setting

**DOI:** 10.1186/s12885-020-06984-1

**Published:** 2020-06-01

**Authors:** Michal Devecka, Marciana Nona Duma, Jan J. Wilkens, Severin Kampfer, Kai Joachim Borm, Stefan Münch, Christoph Straube, Stephanie E. Combs

**Affiliations:** 1 Klinikum rechts der Isar, Department of RadiationOncology, Technical University Munich, Ismaninger Strasse 22, 81675 München, Germany; 2 Department of Radiotherapy and Radiation Oncology, University Hospital of the Friedrich Schiller University, Jena, Germany; 3 Deutsches Konsortium für Translationale Krebsforschung (DKTK)-Partner Site Munich, Munich, Germany; 4 Institute of Innovative Radiotherapy, Helmholtzzentrum München, Munich, Germany

**Keywords:** Cranio-spinal irradiation, Prognostic score, Leptomeningeal carcinomatosis

## Abstract

**Background:**

The aim of our study was to assess the feasibility and oncologic outcomes in patients treated with spinal (SI) or craniospinal irradiation (CSI) in patients with leptomeningeal metastases (LM) and to suggest a prognostic score as to which patients are most likely to benefit from this treatment.

**Methods:**

Nineteen patients treated with CSI at our institution were eligible for the study. Demographic data, primary tumor characteristics, outcome and toxicity were assessed retrospectively. The extent of extra-CNS disease was defined by staging CT-scans before the initiation of CSI. Based on outcome parameters a prognostic score was developed for stratification based on patient performance status and tumor staging.

**Results:**

Median follow-up and overall survival (OS) for the whole group was 3.4 months (range 0.5–61.5 months). The median overall survival (OS) for patients with LM from breast cancer was 4.7 months and from NSCLC 3.3 months. The median OS was 7.3 months, 3.3 months and 1.5 months for patients with 0, 1 and 2 risk factors according to the proposed prognostic score (KPS < 70 and the presence of extra-CNS disease) respectively. Nonhematologic toxicities were mild.

**Conclusion:**

CSI demonstrated clinically meaningful survival that is comparable to the reported outcome of intrathecal chemotherapy. A simple scoring system could be used to better select patients for treatment with CSI in this palliative setting. In our opinion, the feasibility of performing CSI with modern radiotherapy techniques with better sparing of healthy tissue gives a further rationale for its use also in the palliative setting.

## Background

Tumor spread to the leptomeninges (leptomeningeal metastases (LM)) poses a serious condition which leads to rapid deterioration and is ultimately associated with dismal prognosis. Neoplastic meningitis occurs in 3–5% of patients with cancer [1]. Around 70% of LM usually presents in patients with widely-metastatic and progressive cancer, however in 5–10% LM can be the only manifestation of cancer [2]. Sometimes, LM can be the only presentation of the malignant disease. The most common tumors that present with LM are breast cancer, non-small cell lung cancer (NSCLC), and melanoma [3, 4]. Further, some lymphomas have a high risk of CNS dissemination [5].

Current National comprehensive cancer network (NCCN) guidelines recommend that patients with good performance status (Karnofsky performance score (KPS) ≥60) without major neurological deficits, minimal extra-CNS disease, and reasonable systemic options should be treated with induction intrathecal (IT) chemotherapy. In the case of breast cancer primary high dose methotrexate (HD-MTX) can be used. Also, whole brain radiotherapy (WBRT) and radiotherapy (RT) to bulky sites are indicated [6].

European society for medical oncology (ESMO) guidelines recommends consideration of focal RT for circumscribed, symptomatic lesions and WBRT for extensive nodular or symptomatic linear LM. According to ESMO “craniospinal irradiation (CSI) is rarely an option for adult patients with LM from solid cancers because of the risk of bone marrow toxicity, enteritis and mucositis, and the usual co-existence of extra-CNS disease” [7]. Nonetheless, modern radiotherapy techniques such as intensity modulated radiotherapy (IMRT) or proton therapy can reduce the aforementioned toxicities [8–10].

In Germany, the guidelines recommend the use of extended WBRT with the inclusion of the upper two cervical vertebrae and focal RT for bulky disease sites. Multiple factors such as the extent of extracranial disease, and of the LM itself (whether microscopic or macroscopic), patient’s symptoms, KPS, and tumor histology need to be assessed as to what treatment (IT or systemic chemotherapy or radiotherapy) should be used [11].

Hence, though CSI is a mainstay of curative treatment in patients with medulloblastoma and primitive neuroectodermal tumor (PNET), as well as in ependymoma and germinoma with LM, CSI is not yet fully recommended for palliative treatment in all patients with LM.

The aim of our study was to assess the feasibility and oncologic outcomes in patients treated with craniospinal irradiation (CSI) in patients with leptomeningeal metastases (LM) and to develop a pragmatic prognostic score to stratify patients in this palliative setting.

## Methods

### Study patients

Nineteen patients [12] were treated with palliatively intended CSI in our institution between 2001 and 2015. CSI was either performed in one treatment course, or as spinal irradiation (SI) in patients who had already undergone WBRT. A small gap between the preceding WBRT treatment fields was made to avoid overdose from the matched spinal treatment field. Demographic data, as well as histology of the primary tumor, are summarized in Table 1. In order to avoid selection bias all patients who received at least one fraction of CSI or SI were included in our analysis, even though in some case the treatment had to be stopped early.

**Table 1:** Patients’ characteristics (*n* = 19)

Median Age (years)	57.8	(range 31–80)
Median Karnofsky performance index	70	(range 40–90)
Sex	(n)	(percent)
- Male	8	42.1
- Female	11	57.9
Treatment technique	(n)	(percent)
- 2D	3	15.8
- HT	16	84.2
Treatment Field	(n)	(percent)
- CSI	15	78.9
- SI	4	21.1
CNS disease	(n)	(percent)
- Macroscopic	18	94.7
- Microscopic	1	5.3
Presence of systemic disease outside of the CNS	(n)	(percent)
- Yes	7	36.8
- No	11	57.9
- Not available	1	5.3
Primary Diagnosis		
- Breast cancer	5	26.3
- NSCLC	5	26.3
- Non-Hodgkin Lymphoma	3	15.9
- Adenocarcinoma of gastro-esophageal junction	1	5.3
- Astrocytoma WHO Grade III	1	5.3
- Gastric carcinoma	1	5.3
- Malignant peripheral nerve sheath tumor	1	5.3
- non-CNS NGGCT	1	5.3
- Sarcomatoid CUP	1	5.3

Patients treated before 2007 were simulated on a standard treatment simulator (Simulix Evolution, Nucleotron/Elekta). They were immobilized using a thermoplastic mask and vacuum cushion in prone position. Then a 2D treatment plan was calculated using 2 standard opposed fields for the brain with two attached dorsal fields for the spine. All other patients from 2007 on were treated with a helical tomotherapy (HT) Hi-Art machine (Accuracy Inc., Madison, WI, USA). For patients treated with helical tomotherapy (HT), CT imaging was performed with a 3-5 mm slice thickness on a standard Siemens CT (Siemens Inc., Erlangen, Germany). Patients were immobilized in the supine position using a vacuum couch and a thermoplastic head mask (BRAINLAB, Munich, Germany). OARs and PTV were delineated according to institutional guidelines either in iPlan (BRAINLAB, Munich, Germany) or in Eclipse (Varian Medical Systems, Palo Alto, CA, USA). The CTV comprised the craniospinal axis including the nerve root areas and was divided in CTV Brain, CTV cervical spine, CTV thoracic spine and CTV lumbosacral spine until the second sacral vertebra.

The CTV to PTV margins were as follows:

1. CTV Brain: 6 mm–10 mm in all directions
2. CTV cervical spine: 6 mm–10 mm in all directions,
3. CTV thoracic spine: 10 mm anteroposterior and 10-15 mm lateral
4. CTV lumbosacral: 10 mm anteroposterior and 10-20 mm lateral.

The treatment planning was performed with Tomotherapy Planning Station (Tomotherapy Inc., Madison, USA).

The extent of extra-CNS disease was defined by staging CT-scans before the initiation of the CSI (or SI). We reviewed all available MRI findings and images in order to assess the extent of the CNS disease (macroscopic vs. microscopic). All but one patient with breast cancer had positive MRI findings, in one patient the diagnosis was made solely based on spinal tap.

### Assessment of the treatment benefit

The treatment benefit was assessed either clinically (by the improvement in pain, neurological deficits, performance status) or with a proven radiological or CSF response. During the CSI, all patients were seen at least twice a week by a radiation oncologist. First follow up was scheduled in 6 weeks after completion of the treatment and thereafter in 3 months intervals. Follow up consisted of clinical examination and MRI or CSF taps were performed at the discretion of the treating physician.

### Statistical methods

Descriptive statistics were used, to sum up, the demographic and dosimetric information. For survival estimation, the Kaplan-Meier method was used. The overall survival (OS) was calculated from the RT begin to the date of death or censured to the date of the last follow up. Mantel-Cox method was used for the subgroup analysis. All statistical calculations were done in SPSS 23.0 and MS Excel.

### Compliance with ethical standards

This study was approved by the Ethics Committee of the School of Medicine of the Technical University of Munich (number 84/15).

## Results

### Treatment

The median time between primary diagnosis of the disease and the initiation of CSI/SI was 1.16 years (range 0–23.66 years). Ten patients received standard chemotherapy within 3 months prior to the CSI. Three out of these ten patients, received intrathecal chemotherapy (IT) alone. The median total CSI dose was 30.6 Gy (range 3.0–36.0 Gy) with a median dose per fraction 1.6 Gy (range 1.5–1.8 Gy). Fourteen patients received an additional boost to the area of the macroscopic tumor, with a total median dose to the boost area of 37.6 Gy (range 9.0–54.0 Gy). Boost dose per fraction varied between 1.8–3 Gy; one patient had a radiosurgery boost to four cerebral metastases with 16 Gy. No concomitant systemic therapy was given parallel to radiotherapy. All but three patients received dexamethasone during CSI/SI. Three patients received systemic therapy after the CSI/SI. One with mantel cell lymphoma received treatment with Bortezomib for a short period of time which was stopped due to thrombopenia. Patient with hormone-sensitive breast cancer received anastrozole and was alive without signs of disease progress at last follow-up. Another patient with breast cancer responded first to the treatment but was diagnosed with relapse disease 4 months later and received salvage treatment with liposomal cytarabine. She succumbed to disease 5 months later. None of the patients received small molecules, TKIs or immune checkpoint inhibitors prior to the treatment with CSI or thereafter except for bortezomib as stated above.

### Survival and improvement of Symtpoms

Median follow-up and overall survival (OS) for the whole group was 3.4 months (range 0.5–61.5 months). The median OS for patients with LM from breast cancer was 4.7 months and from NSCLC 3.3 months.

**Fig. 1:**
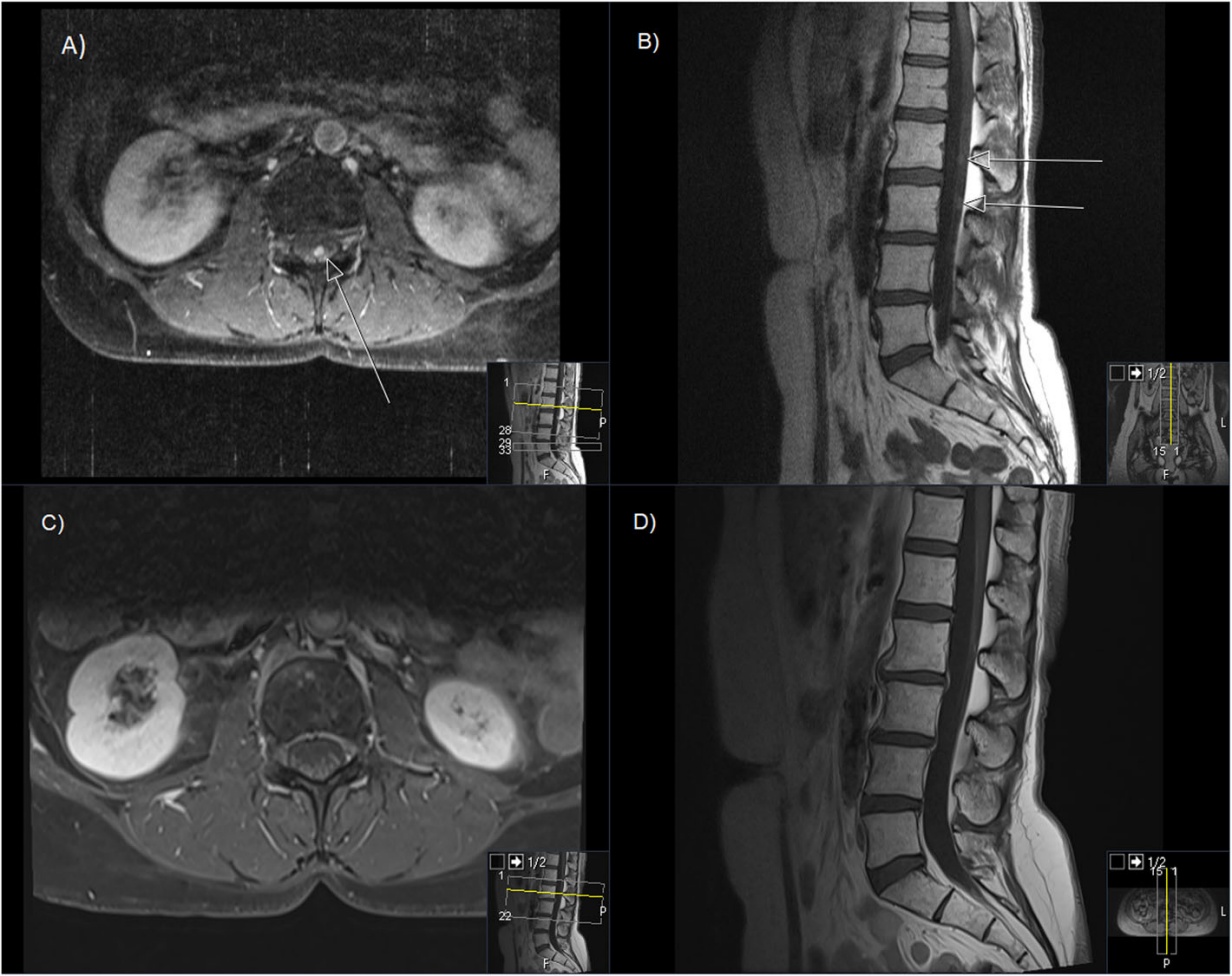
Example of a patients’ MRI before and 6 months after CSI. Contrast enhanced T1 MRI sequence A,B) Prior to CSI, C,D) 6- months post CSI; A,C) axial view B,D) sagittal view. Arrows point at macroscopic intraspinal disease

11 out of 19 patients benefited from the treatment either clinically (by the improvement in pain, neurological deficits, performance status) or with a proven radiological or CSF response. There were four “long-term” survivors: two breast cancer patients surviving for a minimum of 11.3 (alive at last follow-up) and 13 months respectively, one patient with disseminated astrocytoma WHO grade III surviving 16 months (alive at last follow-up) and one patient with NSCLC surviving for more than 5 years (alive at last follow-up). An example of one of these patients is shown in Fig. 1. In 10 out of 19 patients a neurological benefit or pain reduction was recorded after CSI/SI. One patient had no neurologic symptoms prior to treatment or therafter. In 8 out of 19 patients no improvement was observed (Table 2).

**Table 2:** Patients’ treatment and outcome

Patient	Primary diagnosis	Age at treatment	Treatment	CSI/SI Dose (Gy)	CSI/SI Dose per Fraction (Gy)	Boost Region	Cumulative dose in boosted volume (Gy)	Boost Dose per Fraction (Gy)	Reason for treatment discontinuation	Clinical treatment benefit	Prognostic Score	Survival (months)	Patient alive at last follow up
1	NSCLC	64	CSI	20,8	1.6	no Boost	NA	NA	clinical progress, deterioration of physical condition	No	2	1.5	No
2	Breast cancer	37	CSI	35.2	1.6	Posterior fossa	50.0	2.0	NA	No	1	3.8	No
3	Breast cancer	58	CSI	18.0	1.8	A) Sacrl cauda equina B) whole brain - C7	A)26.0 B) 37.0	A) 2 × 2.0 B) 2 × 2.0 + 5 × 3.0	Grade III Leuko- and Thrombopaenia	Improvement in paresis, headaches and dizziness	0	4.7	No
4	NSCLC	70	CSI	36.0	1.8	Sacrum	43.5	2.5	NA	Pain reduction	1	3.3	No
5	Breast cancer	63	CSI	21.6	1.8	Radiosurgery Boost to four brain metastases	37.6	16.0	Grade III Leuko- and Thrombopaenia	No	1	3.3	No
6	Primary CNS Lymphoma	67	SI	21.0	1.5	L3-S1, whole brain	30.0	3.0	Grade III Leuko- and Grade IV Thrombopaenia	No	0	1.9	No
7	DLBCL	64	SI	3.0	1.5	Th8 - Sacrum	9.0	3.0	Grade IV Leuko- and Grade III Thrombopaenia	No	1	0.5	No
8	NSCLC	80	CSI	30.6	1.8	Th11-S2	40.6	2.0	clinical progress, deterioration of physical condition	No	2	1.5	No
9	NSCLC	48	CSI	36.0	1.5	no Boost	NA	NA	NA	Pain reduction	0	61.5	Yes
10	Anaplastic Astrocytoma	63	CSI	36.0	1.8	A) C2-C6,Th1-Th2, Th6-Th10 B) left cerebellum	A) 45.0 B) 54.0	1.8	NA	Improvement of dizziness	0	16.3	Yes
11	Sarcomatoid CUP	41	CSI	23.6	1.6	no Boost	NA	NA	clinical progress, deterioration of physical condition	No	1	1.6	No
12	Adenocarcinoma of Gastroesophagheal Junction	48	CSI	14.8	1.8	C6, Th5–6, L1–5	24.8	2.0	clinical progress, deterioration of physical condition, massive thrombosis	No	2	0.9	No
13	Gastric Cancer	36	CSI	30.0	1.5	Posterior fossa, TH7	36.0	1.8	NA	Improvement of blurred vision and paraesthesia and ataxia	0	3.4	No
14	Embryonal Carcinoma (NOS)	47	SI	32.0	1.6	no Boost	NA	NA	NA	Improvement of Paresis	2	2.8	No
15	MPNST	31	CSI	35.2	1.6	Posterior fossa	54.0	2.0	NA	Improvement in general physical condition, better mobilisation, pain reduction	1	8.4	No
16	Mantel Cell Lymphoma	70	CSI	28.8	1.8	no Boost	NA	NA	Grade IV Leukopaenia	No but negativ spinal tap one month after CSI	0	7.3	No
17	NSCLC	56	SI	36.0	1.8	A) Th9–10 B) L3 -S2	A) 44.0 B) 50.0	A) 2.2 as simultaneuous integrated boost B) 2.2 as simultaneuous integrated boost followed by 2 × 3.0	NA	Improvement of bladder incontinence and paresis	1	4.2	No
18	Breast Cancer	69	CSI	36.0	1.8	no Boost	NA	NA	NA	Almost complete regression of sensory and motor deficits in the lower left extremity as well as complete regresion of the impairment in urinary and defecation function	0	11.3	Yes
19	Breast Cancer	36	CSI	35.2	1.6	no Boost	NA	NA	NA	Improvement in general clinical condition, pain and sensitive neurological deficits	0	13.0	No

**Fig. 2:**
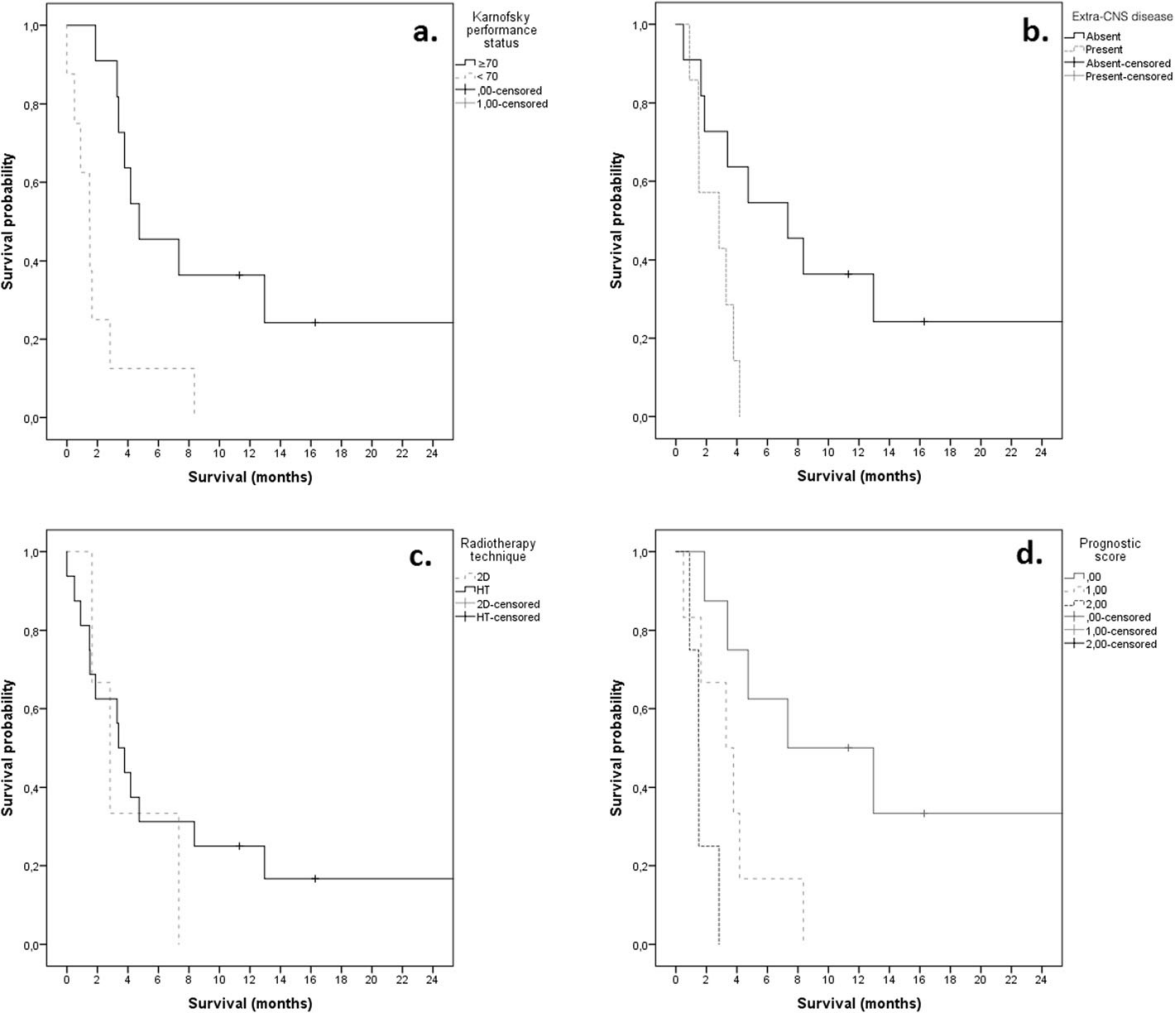
Survival Probability according to KPS, extra-CNS disease, radiotherapy technique and estimated prognostic score

In the subgroup analysis (univariate and multivariate) of our group, performance status and extra-CNS disease were significantly associated with survival. Patients with KPS ≥70 (*p* = 0.018) and no extra-CNS disease (*p* = 0.032) fared much better than their counterparts (Fig. 2). The RT technique had no significant impact on survival (*p* = 0.944; Fig. 2).

A prognostic score combining the KPS and the presence of extra-CNS disease is suggested to stratify patients with LM (Table 3). In our patients’ cohort, the median OS was 7.3 months, 3.3 months and 1.5 months for patients with 0, 1 and 2 risk factors (KPS < 70 and the presence of extra-CNS disease) respectively. The prognostic score combining these two factors is highly significant (*p* = 0.001) (Fig. 2).

**Table 3:** OS according to prognostic score based on KPS and presence of extra-CNS disease

Score		Median OS (months)	Median OS (weeks)
0	No extra-CNS disease and KPS ≥ 70	7.3	31.9
1	One of the two factors present	3.3	14.3
2	Extra-CNS disease present and KPS < 70	1.5	6.4

### Side effects

Nine patients did not complete the prescribed treatment. The reasons for treatment discontinuation were mostly grade III-IV cytopenias (5 patients) not recovering after treatment break or general status deterioration with progressive disease in spite of the treatment (4 patients). One patient died of massive thrombosis 1 week after the treatment was discontinued. As his treatment was discountinued due to grade II thrombopaenia (62,000 Tro/μl) this grade V toxicity was most likely unrelated to the CSI. Hematologic toxicities are summarized in Table 4. Other side effects included xerostomia (*n* = 3), dysgeusia (*n* = 2), candida infection (n = 2), nausea (*n* = 4), vomiting (*n* = 1), mucositis/dysphagia (*n* = 5). All of these side effects were mild (grade I-II).

**Table 4:** Hematologic toxicity according to CTCAE v 4.03

Grade	0	1	2	3	4
Anemia	12 (63.2%)	7 (36.8%)	–	–	–
Leukopenia	3 (15.8%)	4 (21.1%)	5 (26.3%)	5 (26.3%)	2 (10.3%)
Thrombopenia	1 (5.3%)	6 (31.6%)	5 (26.3%)	6 (31.6%)	1 (5.3%)

## Discussion

In our palliative treatment group, the OS of patients receiving CSI due to LM was 3.4 months (14.7 weeks; range 4–267 weeks). This is in line with survival times reported by the literature on either IT or systemic chemotherapy where median OS ranges between 7 and 30.3 weeks (Table 5).

**Table 5:** Overview of literature on CSI

Trial	Design	Outcome
Grossman et al.	IT MTX versus thiotepa (59 patients; solid tumors and lymphoma)	OS, 15.9 (MTX) versus 14.1 weeks (thiotepa)
Hitchins et al.	IT MTX versus MTX + CYT (44 patients; solid tumors and lymphoma)	OS, 12 (MTX) versus 7 weeks (MTX + CYT)
Glantz et al.	LS-CYT versus MTX (61 patients; solid tumors)	OS, 105 (LS-CYT) versus 78 days (MTX), difference not significant
Glantz et al.	LS-CYT versus CYT (28 patients; lymphoma)	OS, 99.5 (LS-CYT) versus 63 days (CYT), difference not significant.Cytologic response rate 71% (LS-CYT) versus 15%
Boogerd et al.	IT versus no IT therapy, but systemic therapy and RT were given in both arms (35 patients; breast cancer)	OS, 18.3 (IT) versus 30.3 weeks (no IT)
Shapiro et al.	Lymphoma (25 patients)	LS-CYT versus all MTX and CYT-treated patients combined: PFS 35 versus 43 days (not significant)
		LS-CYT versus MTX: PFS 35versus 37.5 days (notsignificant)

From Leal at al. Abbreviations: IT intrathecal, LM leptomeningeal metastasis, MTX methotrexate, CYT cytarabine, LS-CYT liposomal cytarabine, PFS progression-free survival, RT radiotherapy. (Leal 2011)

Focal RT either for bulky spinal disease or WBRT remains a backbone of treatment of patients with LM even though its effect on survival can be questioned [13].

Boogerd et al. showed in a randomized trial that the addition of intrathecal chemotherapy to systemic chemotherapy and focal RT did not provide any additional survival benefit and led to increased toxicity in patients with LM form breast cancer [14]. Also, in the retrospective study by Oechsle et al. systemic chemotherapy resulted in significantly improved OS as compared to patients treated only with intrathecal chemotherapy, RT or both. Patients who got systemic chemotherapy had a median survival of 24.9 weeks. However, in this study, solid tumors accounted only for 54% and the rest were hematological malignancies, which normally respond better than solid tumors to treatment with systemic chemotherapy [15]. A number of other reports showed the efficacy of systemic agents (e.g. temozolomide, capecitabine). In some cases even durable responses were shown [13, 16–19].

Pan et al. tested a regimen of intrathecal chemotherapy with MTX and involved field radiotherapy (whole brain or focal to spinal lesions) with 40 Gy in 2 Gy/fraction. Fifty-nine patients were treated with a median OS of 6.5 months [12].

There is very limited data on the use of CSI as a treatment for patients with LM. To our knowledge, there are only two published study where CSI was evaluated in a palliative setting similar to our study [20, 21].

Hermann et al. studied 16 patients with LM from solid tumors. Nine breast cancer, five NSCLC, one with cancer of unknown primary (CUP) and one renal cell cancer patients were included in his study. The median OS for all patients was 12 weeks (range 4–84 weeks). For patients treated with CSI, only the median OS was 8 weeks. The median OS was 16 weeks for those treated with IT chemotherapy and CSI. Nonetheless, all patients had synchronous extra-CNS metastases in this study. Improvement in symptoms was seen in 11 patients, two patients had stable disease and in three patients therapy was ended prematurely, due to progressive disease [20]. However, in this study patients were treated without the use of modern RT techniques.

In a recent published study, El Shafie et al. reported on 25 patients treated for LM with the use of HT. The prescribed dose was 36Gy in 20 fractions. Sixteen patients had metastatic disease outside of the CNS and 18 had parenchymal brain metastases. Majority of the patients had breast (*n* = 15) or lung cancer (*n* = 6). Median OS from the diagnosis of LM was 19.3 weeks. In multivariate analysis KPS ≥ 70, neurologic response and age < 55 years were prognostic for improved OS [21].

In the analysis of our palliative patient cohort, patients having KPS ≥ 70 fared also significantly better. For patients with KPS ≥ 70, the median OS was 4.7 months (20.6 weeks) compared with 1.5 months (6.6 weeks) for the rest of the group. Further, patients having no extra-CNS disease showed significantly improved survival with a median OS for patients with the leptomeningeal disease only of 7.3 months (31.9 weeks) as compared to 2.8 months (12.3 weeks) for those also having systemic extra-CNS disease. When these factors were combined a median OS of 7.3 months (31.9 weeks) could be achieved for those who had a good KPS and no extra-CNS disease. In those who had one or two adverse factors, the median OS was 3.3 months (14.3 weeks) and 1.5 months (6.4 weeks) respectively. These results suggest that cautious patient selection is needed in a palliative setting when considering CSI. Despite the drop-out rate of our patients’ collective 11/19 which was much higher than that in the study by Hermann the patients have profited from the treatment either for survival prolongation or for symptom control.

CSI is rarely considered as a treatment option for patients with the leptomeningeal metastatic disease. However, our study together with the study by El Shafie et al. gives a good rationale for offering CSI to well-selected patients. Especially those who have a good performance status, and present with none or controlled extra-CNS disease seem to have the best prognosis after CSI. Although the study by Hermann et al. suggested that patients receiving IT chemotherapy before the CSI fare better, it is somehow counterintuitive first to use IT chemotherapy and to perform radiotherapy after that. If radiation can achieve limited control of macroscopic disease, it is probably moreover so capable of controlling microscopic disease (e.g., the free tumor cells in liquor which could have been affected by the intrathecal chemotherapy). So perhaps intrathecal chemotherapy could be used after prior CSI as “adjuvant” therapy. There are no studies to support this, and there is much fear to apply methotrexate after CSI due to its side effect, but perhaps CSI followed by liposomal cytarabine would be an interesting approach which requires further evaluation.

Historically, two major reasons for the underuse of CSI in patients with LM existed. The concern for acute side effects and technically difficult treatment application. In the Hermann et al. study the major toxicities were myelosuppression (69%), dysphagia (56%), mucositis (44%) and nausea (19%). No grading of these toxicities was reported [20]. In the study by El Shafie mild (CTCAE Gr I-II) fatigue and nausea was reported in 84 and 36% respectively. Skin erythema appeared in 28% and myelosupression in 32%. Five patients did not complete the prescribed treatment [21]. In our study, the non-hematological toxicities were mild (Grade I-II) and consisted of xerostomia 16%, dysgeusia 10%, nausea 21% with vomiting in 5% and mucositis/dysgeusia in 26% with mucosal candidosis in 10% patients. Also, most of our patients suffered from hematological toxicity. In our collective, there was a big drop-out rate as 47.4% of the patients did not complete their prescribed treatment, due to progressive disease or major cytopenia. Nonetheless, with modern radiotherapy techniques CSI can be applied with limited acute non-hematological toxicities [8, 22–25]. Further, when using helical Tomotherapy (HT), there is no need for field junctions and their daily or weekly shifts as it was the case in the 2D/3D era.

Hence these historical reasons apply only to a certain extent in the modern-day radiation oncology. CSI targets all of the leptomeningeal metastatic disease, macroscopic and microscopic. Patients receiving CSI do not have a risk of developing radiculitis, meningitis, and several other side effects and they are not subjects of repeated lumbar punctures which are needed to apply IT chemotherapy and are themselves painful and hence decrease the quality of life.

## Limitations

The major limitations of our study was its retrospective nature, the patients’ selection bias, and small and heterogeneous patients’ collective. All patients were treated before 2016 and none of the patients received small molecules, TKIs or immune checkpoint inhibitors prior to the treatment with CSI or thereafter. However, these of novel agents are used more frequently today may have an impact on oncologic outcome after CSI. Furthermore, due to the retrospective design, there is a potential selection bias regarding which patients have received a CSI.

## Conclusion

CSI demonstrated clinically meaningful survival that is comparable to the reported outcome of intrathecal chemotherapy. A simple scoring system could be used to better select patients for treatment with CSI in the palliative setting. Especially in patients with larger macroscopic lesions, the benefit of RT is undisputed. In our opinion, the feasibility of performing CSI with modern radiotherapy techniques with better sparing of healthy tissue gives a further rationale for its use also in the palliative setting.

## Data Availability

The datasets used and/or analyzed during the current study are available from the corresponding author on reasonable request.

## Abbreviations

LM: leptomeningeal metastases
NSCLC: non-small cell lung cancer
NCCN: National comprehensive cancer network
KPS: Karnofsky performance score
IT: intrathecal
HD-MTX: high dose methotrexate
WBRT: whole brain radiotherapy
RT: radiotherapy
ESMO: European society for medical oncology
CSI: craniospinal irradiation
IMRT: intensity modulated radiotherapy
PNET: primitive neuroectodermal tumor
SI: spinal irradiation
HT: helical tomotherapy
OAR: organs at risk
CTV: clinical target volume.
PTV: planning target volume
OS: overall survival
